# Selective targeting of MD2 attenuates intestinal inflammation and prevents neonatal necrotizing enterocolitis by suppressing TLR4 signaling

**DOI:** 10.3389/fimmu.2022.995791

**Published:** 2022-11-01

**Authors:** Dabin Huang, Ping Wang, Juncao Chen, Yanbin Li, Mingwei Zhu, Yaping Tang, Wei Zhou

**Affiliations:** ^1^ Department of Neonatology, Guangzhou Women and Children’s Medical Center, Guangzhou Medical University, Guangzhou, China; ^2^ Department of Pediatrics, Guangdong Second Provincial General Hospital, Guangzhou, China; ^3^ Guangzhou Institute of Pediatrics, Guangzhou Women and Children’s Medical Center, Guangzhou Medical University, Guangzhou, China

**Keywords:** NEC, MD2, TLR4, NF-κB, intestinal barrier

## Abstract

Neonatal necrotizing enterocolitis (NEC) is an inflammatory disease that occurs in premature infants and has a high mortality rate; however, the mechanisms behind this disease remain unclear. The TLR4 signaling pathway in intestinal epithelial cells, mediated by TLR4, is important for the activation of the inflammatory storm in NEC infants. Myeloid differentiation protein 2 (MD2) is a key auxiliary component of the TLR4 signaling pathway. In this study, MD2 was found to be significantly increased in intestinal tissues of NEC patients at the acute stage. We further confirmed that MD2 was upregulated in NEC rats. MD2 inhibitor (MI) pretreatment reduced the occurrence and severity of NEC in neonatal rats, inhibited the activation of NF-κB and the release of inflammatory molecules (TNF-α and IL-6), and reduced the severity of intestinal injury. MI pretreatment significantly reduced enterocyte apoptosis while also maintaining tight junction proteins, including occludin and claudin-1, and protecting intestinal mucosal permeability in NEC rats. In addition, an NEC *in vitro* model was established by stimulating IEC-6 enterocytes with LPS. MD2 overexpression in IEC-6 enterocytes significantly activated NF-κB. Further, both MD2 silencing and MI pretreatment inhibited the inflammatory response. Overexpression of MD2 increased damage to the IEC-6 monolayer cell barrier, while both MD2 silencing and MI pretreatment played a protective role. In conclusion, MD2 triggers an inflammatory response through the TLR4 signaling pathway, leading to intestinal mucosal injury in NEC. In addition, MI alleviates inflammation and reduces intestinal mucosal injury caused by the inflammatory response by blocking the TLR4-MD2/NF-κB signaling axis. These results suggest that inhibiting MD2 may be an important way to prevent NEC.

## Introduction

Neonatal necrotizing enterocolitis (NEC) is a common and serious intestinal inflammatory disease occurring in the neonatal period and is considered a destructive gastrointestinal disorder leading to the death of premature infants (1–3). A cohort study of 9,552 premature infants with gestational age less than 32 weeks found that the incidence of NEC was 4.9% based on data collected from 57 tertiary hospitals in 25 provinces of China in 2019 (4). The common long-term and severe complications of the NEC survivors included delayed neuropsychomotor development, short bowel syndrome, cholestasis, intestinal failure and intestinal stenosis (5–7). Although NEC etiology and pathogenesis are not clearly understood, studies have shown that exaggerated inflammatory signal imbalance in the intestinal mucosa and intestinal injury work together to induce NEC in premature infants (8). Pattern recognition receptors are widely expressed in various cells of the intestinal epithelium; these receptors recognize pathogenic molecules and immunogenic factors and initiate a series of signal cascade reactions. Thus, pattern recognition receptors are the key component of intestinal innate immunity and play a central role in the first line of intestinal immunity defense (9, 10). Toll-like receptors (TLRs) are a class of typical pattern recognition receptors (8–10). As microbial ligands stimulate intestinal mucosa, TLR inflammatory signaling pathways are activated to begin the inflammatory cascade. In particular, TLR4, which is expressed in intestinal epithelial cells, is the receptor for lipopolysaccharide (LPS), and is associated with the occurrence and development of NEC (11–14). Exaggerated proinflammatory signal transduction of the TLR4 signaling pathway promotes mucosal injury in the intestine and enhances the destruction of the intestinal barrier (15). TLR4-induced epithelial cell death in the intestine is the foundation for intestinal mucosal injury in NEC (16–18). Targeting the TLR4 signaling pathway might be an attractive approach for treating NEC based on studies linking TLR4 and NEC.

As a coreceptor of TLR4, myeloid differentiation protein 2 (MD2) is required in order to recognize LPS and subsequently activate the TLR4 signaling pathway (19, 20). When LPS binds to MD2, the LPS-MD2-TLR4 complex is dimerized and further recruits myeloid differentiation factor 88 (MyD88). This event leads to activation of downstream signaling pathways, especially the nuclear factor kappa-B (NF-κB) pathway, which results in the expression of proinflammatory molecules (20). A clinical study found that the transcription level of the MD2 gene in acute-phase intestinal tissues of NEC patients was approximately 8 times higher than that in recovery-phase tissues (21). A study of 42 newborns with NEC showed that a polymorphic site in the promoter region of MD2 (rs11465996) was closely associated with the occurrence and severity of NEC (22). Previous studies have shown that human milk oligosaccharides can reduce the severity of NEC in mice and piglets, and computer molecular modeling suggested that human milk oligosaccharides might compete for the LPS binding sites on the MD2-TLR4 complex (23). Therefore, MD-2 may be related to the development of NEC, and its abnormal expression, structural abnormalities, or functional abnormalities may affect the expression of the MD2-TLR4/NF-κB signaling pathway and cause an abnormal immune response. The LPS-induced inflammatory response may be significantly diminished by targeting MD2 (24), thereby decreasing the likelihood of adverse events including sepsis (25), cardiac/kidney injuries (26, 27), cancer (28), colitis (29) and neuroinflammation (30). These findings indicate that targeting MD2 to inhibit the excessive activation of TLR4 signaling may be an effective strategy for the treatment of NEC.

At present, the role of MD2 in NEC is unknown. Our clinical samples showed that MD2 expression was significantly higher in acute-phase intestinal tissue of NEC patients than in tissue collected in the recovery period. We hypothesized that injury to intestinal epithelial cells induced by the TLR4 signaling pathway is dependent on MD2. In this study, an MD2 inhibitor (MI) was applied to an NEC rat model, and different MD2 intervention strategies (MD2 overexpression, MD2 knockout and MI pretreatment) were used in IEC-6 enterocytes to test this hypothesis. Overall, we elucidated the mechanism of MD2 in the occurrence and development of NEC. Further, the results suggested that MD2 may be an effective target for reducing the incidence and severity of NEC by early diagnosis and intervention.

## Materials and methods

### NEC patient selection and sample collection

According to the Declaration of Helsinki, after informed consent from the patients’ parents in Department of Neonatology in Guangzhou Women and Children’s Medical Center and approval by the Medical Ethics Review Committee were obtained (Ethical approval number: [2022] 059A01), the clinical intestinal tissues of NEC neonates and control neonates were collected. The intestinal tissue of NEC neonates came from patients who underwent intestinal resection due to severe intestinal necrosis. The severity of NEC was assessed in clinics based on Bell’s classification, which identifies the disease as stage I, II, or III (31). In our study, we collected samples from 4 infants with Bell stage II and III NEC, 2 convalescent infants with NEC and 2 infants with congenital intestinal atresia as controls. The inclusion criteria for the subjects in the control group were based on previous studies (32).

### Animal experiments

All animal experiments were approved by the Institutional Animal Care and Use Committee of Guangzhou Medical University and performed according to the institutional guidelines. Forty-eight newborn male or female Sprague–Dawley (SD) rats (6–8 g) were collected from pregnant SD rats (Beijing Vital River Laboratory Animal Technology Co., Ltd., Beijing, China). Neonatal rats were separated from maternal rats immediately after birth and randomly divided into 4 groups with 12 rats in each group. The overall experimental scheme, including grouping, NEC protocol, treatment and sampling times, is summarized in **sFigure 1**.

The NEC model was achieved by formula feeding and the asphyxia/cold stress method with reference to previous studies (33, 34). Briefly, a newborn incubator was used to warm and humidify neonatal rats at 37°C. After stabilization, rats were hand-fed with formula gavage (Abbott Similac infant formula: Esbilac canine milk replacer 2:1) six times per day. A slow increase in feeding was introduced at the onset, starting at 0.10 mL every four hours and gradually increasing to 0.2–0.3 mL every four hours. In addition, every 12 hours, the neonatal rats were subjected to hypoxia (95% nitrogen for 90 seconds) and hypothermia (4 °C for 10 min). In the treatment group, rats were given MI (20 mg/kg/d) by gavage (35). MI (MD2-IN-1, Selleck, Shanghai, China) was dissolved in 1% sodium carboxymethyl cellulose (CMC-Na) for *in vivo* administration. MD2-IN-1 is a specific MD2 inhibitor that blocks the LPS-induced activation of TLR4/MD2-downstream proinflammatory MAPK/NF-κB signaling pathways by binding to the hydrophobic pocket of MD2 (35). In the control group, rats were normally breast fed. As a vehicle control, animals were gavaged with 1:100 dilutions of CMC-Na. Throughout the experiment, the clinical signs of NEC, such as abdominal distension, stool traits, gastric retention, apnea and lethargy, were monitored every 6 hours in the animals. Animals were euthanized after 72 hours or when clinical symptoms of NEC appeared.

### Animal sampling

On the 7th day after birth, the rats were euthanized after fasting for 12 hours, and the intestines were collected and evaluated macroscopically for signs of NEC (gas accumulation, hemorrhage and necrosis). Upon sacrifice, the ileum was taken from the ileocecum junction approximately 1 cm away, fixed with 4% paraformaldehyde and embedded in paraffin for histological examination or stored at -80° for protein collection and cytokine measurement.

### Histopathological examination and grading

Paraffin ileal tissue sections (4 mm) of humans and rats were prepared and stained with hematoxylin and eosin after dewaxing and hydration. The stained sections were observed by optical microscopy. The intestinal injury of rats was evaluated according to the histopathological scoring system of Nadler (36), and the pathological grades were as follows: grade 0 indicates normal intestine; grade 1 indicates exfoliation or separation of epithelial cells; grade 2 indicates moderate submucosal separation and local villous necrosis; grade 3 indicates severe submucosal separation and villous necrosis; and grade 4 indicates transmural necrosis. Samples graded 2 or higher were classified as NEC positive after histopathologic evaluation by two blinded pathologists.

### Western blotting and immunoprecipitation

The total protein from the ileal tissues was extracted with lysis buffer (Beyotime, China). A Nuclear and Cytoplasmic Protein Extraction Kit (Beyotime, China) was used for the preparation of nuclear and cytoplasmic fractions. The concentration of protein was determined by BCA assay (Invitrogen, USA). The protein was separated by SDS−PAGE and transferred to polyvinylidene fluoride membranes to further incubate with 5% skim milk in TBS/0.05% Tween-20 for 1 hour at room temperature. Following overnight incubation with primary antibodies at 4°C, membranes were incubated with HRP-conjugated secondary antibodies for 1 hour. These were the primary antibodies used: anti-MD2 (1:500, ab24182, Abcam), anti-MyD88 (1:1000, NB100-56698, Novus), anti-TLR4 (1:1000, sc-293072, Santa Cruz), anti- inhibitor of IκB alpha (IκBα) (1:1000, 10268-1-AP, Proteintech), anti-Phospho-IκBα (Ser32) (1:1000, 2859, Cellsignal), anti-NF-κB p65 (1:1000, 10745-1-AP, Proteintech), anti-phospho-NF-κB p65 (Ser536) (1:1000, 3033, Cellsignal), anti-high-mobility group box 1 (HMGB1) (1:1000, 10829-1-AP, Proteintech), anti-caspase-3 (1:1000, 19677-1-AP, Proteintech), anti-cleaved-caspase-3 (1:1000, 9661, Cellsignal), anti-occludin (1:1000, 66378-1-Ig, Proteintech), anti-claudin-1 (1:1000, 13050-1-AP, Proteintech), anti-GAPDH (1:2000, 2118, Cellsignal), anti-β-actin (1:2000, 4970, Cellsignal) and anti-Lamin-B1 (1:1000, 12987-1-AP, Proteintech). Immunoreactive bands were detected by enhanced chemiluminescence reagents (Millipore, USA). ImageJ software (National Institutes of Health, Bethesda, MD) was used to calculate relative protein levels.

For coimmunoprecipitation assays, intestinal tissue lysates (300–500 μg) were subjected to an overnight incubation with primary TLR4 antibody at 4°C and then immunoprecipitated using Protein A/G Sepharose beads. The bound protein was eluted in denatured SDS loading buffer and detected by western blotting.

### Immunofluorescence staining

For immunofluorescence, sections of paraffin ileum tissue (4 mm) were prepared and fixed with 4% PFA. Then, 5% goat serum was added for 1 hour at room temperature, and the primary antibodies were applied overnight in a dark chamber at 4°C. After incubating with the second antibody at room temperature for 1 hour, the sections were mounted with VECTASHIELD Antifade Mounting Medium with DAPI to stain the nucleus. Using a Leica SP8 inverted fluorescence microscope, immunofluorescent images were acquired. ImageJ software was used to calculate the average fluorescence intensity of MD2 and occludin.

The following was the primary antibodies and their concentrations: rabbit anti-rat MD2 (ab24182, Abcam) (1:50), mouse anti-rat occludin (66378-1-Ig, Proteintech) (1:200). The secondary antibodies and their concentrations were as follows: goat anti-mouse or rabbit IgG H&L-Alexa Fluor 555 (ab150114 or ab150078, Abcam) (1:1000).

### ELISA

According to the manufacturer’s instructions, ELISA kits (CUSABIO, Wuhan, China) were used to measure IL-6 and TNF-α concentrations in rat intestinal homogenates. The BCA method was used to measure the total protein content.

### Intestinal permeability measurement

After the NEC protocol was performed, rats were gavaged with fluorescein isothiocyanate-dextran (FITC-D, Sigma; MW 10,000; 40 mg FITC-D/100 g) 4 hours before being sacrificed. After collecting the serum of rats, the fluorescence density was measured by a Multiskan Go plate reader (Thermo Fisher, USA). Additionally, the serum FITC-D concentration was determined.

### TUNEL staining

According to the manufacturer’s protocol, the TUNEL assay was performed by using the *In Situ* Cell Death Detection Kit TMR Red (12156792910, Roche). Enterocyte apoptosis *in vivo* was determined by measuring the number of intestinal epithelial cells that were TUNEL^+^ by confocal microscopy per high-power field, and three high-power visual fields were randomly selected for each sample. The area of intestinal tissue in each high-power field was calculated by ImageJ software. To calculate the number of apoptotic cells per square millimeter, we used the following formula: number of apoptotic cells per square millimeter (number/mm^2^) = TUNEL^+^ cell number/area mm^2^.

### Cell culture and treatment

IEC-6 enterocytes were purchased from iCell Bioscience Inc. (Shanghai, China) and cultured according to the manufacturer’s recommendations. DMEM-high glucose medium (GIBCO, USA) containing 10% fetal bovine serum and 1% antibiotics (100 U/mL penicillin and 0.1 mg/mL streptomycin) was used for cell culture. LPS (L2880, Sigma) was added to serum-free DMEM according to the experimental design. MI was dissolved in DMSO for *in vitro* experiments, and cells in the vehicle control were treated with DMSO at a 1:1000 dilution.

### Lentivirus transduction and small interfering RNA transfection

Lentiviral vector particles (Genechem, Shanghai, China) containing MD2 short hairpin RNA (shRNA) were transfected into intestinal cells to generate MD2-overexpressing IEC-6 enterocytes. The shRNA sequence is as follows: 5′-GAG GAT CCC CGG GTA CCG GTC GCC ACC ATG TTG CCA TTT TTT CTC TTT TC-3′. Cell lines overexpressing MD2 were screened using medium containing puromycin (3 μg/mL). An siRNA specific for MD2 (5′-CAU GUU GAG UUC AUU CCA ATT-3′) was designed and synthesized by Tsingke (Beijing, China). According to the manufacturer’s protocol, this siRNA was transfected into cells with Lipofectamine 3000 transfection reagent (Invitrogen, Waltham, MA, USA). MD2 protein and mRNA expression levels were detected by real-time quantitative PCR (RT–qPCR) and western blot in each group after overexpression or knockdown of MD2.

### RT–qPCR

TRIzol reagent (Invitrogen) was used to extract total RNA from enterocytes, after which a PrimeScript RT kit (RR047A, Takara, Japan) was used to reverse transcribe the RNA to cDNA. RT–qPCR was performed using a SYBR Premix Ex Taq II kit (RR820A, Takara, Japan) according to the manufacturer’s protocol. The following is the information of the primers: rat MD2 (forward: 5′-CTG AAA GGG GAG GCT GTC AAC AC-3′; reverse: 5′-GCA ATG GCT TCT GCA ACA CAT CTG-3′); rat TNF-α (forward: 5′-ATG GGC TCC CTC TCA TCA GTT CC-3′; reverse: 5′-GCT CCT CCG CTT GGT GGT TTG-3′); rat IL-6 (forward: 5′-ACT TCC AGC CAG TTG CCT TCT TG-3′; reverse: 5′-TGG TCT GTT GTG GGT GGT ATC CTC-3′); rat beta-actin (forward: 5′-CTG AGA GGG AAA TCG TGC GTG AC-3′; reverse: 5′-AGG AAG AGG ATG CGG CAG TGG-3′).

### Determination of transepithelial electrical resistance and FITC-D permeability measurement

The Transwell cells were inoculated with 5 × 10^4^ cells per well (200 μL) in the upper compartment, and 600 μL culture medium was added to the lower compartment. Transepithelial electrical resistance (TEER) was measured by a Millicell-ERS voltmeter after changing the culture medium and balancing the incubator for 30 minutes. In short, the two electrode probes of the resistance instrument were placed in the upper and lower compartment of the Transwell chamber, and the average TEER values of three different points were measured and calculated as the actual TEER values. The assay was carried out in triplicate. Two Transwell chambers with only culture medium were used as blank controls. To calculate the standard TEER value, we used the formula TEER value = (measured value − blank control value)/0.33 cm^2^.

After relevant intervention, FITC-D (100 μL, 1 mg/mL) was added to the upper compartment of the Transwell chamber, and 600 μL serum-free medium was added to the lower compartment. Then, the Transwell chamber was incubated at 37°C for 1 hour. The medium in the lower compartment was collected, and the fluorescence intensity was measured by a Multiskan Go plate reader (ThermoFisher, USA) (emission wavelength, 525 nm; excitation wavelength, 488 nm). According to the standard curve, the fluorescence intensity of each sample was converted into the FITC-D concentration. To measure FITC-D permeability, the following equation was used: FITC-D permeability (%/h/cm^2^) = (FITC-D concentration in the lower compartment/initial concentration of FITC-D upper compartment)/1 hr/0.33 cm^2^ × 100%.

### Statistical analysis

Data analysis was performed using SPSS 25.0 software (SPSS, Inc., Chicago, IL, USA) and GraphPad Prism 8 (GraphPad Software Inc., USA). The comparison of multiple groups was performed using one-way analysis of variance (ANOVA) followed by Tukey’s *post hoc* test. A two-tailed unpaired t test was used for comparisons between two groups. The pathological scores among multiple groups were compared by the Kruskal–Wallis test. All data are presented as the mean ± standard deviation. P<0.05 was considered a statistically significant difference.

## Results

### Clinical characteristics and MD2 expression levels in intestinal tissues of NEC patients

**Figure 1:**
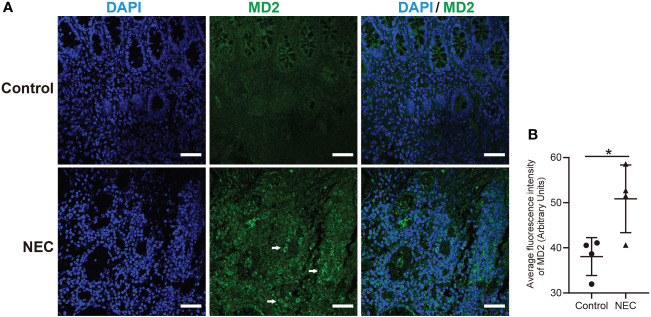
Clinical characteristics and MD2 expression levels in intestinal tissues of NEC patients. **(A)** MD2 expression in intestinal tissue of patients. MD2 (green) and DAPI staining of nuclei (blue) in intestinal tissue of the control and NEC groups. Scale bar, 50 μm. **(B)** Average fluorescence intensity of MD2. Data are presented as the mean ± SD (n=4). The scatter plots represent the mean value of the average intensity of fluorescence in 6 random fields in each section of the quantification diagram. *p value < 0.05 for each group comparison using a two-tailed Student’s t test.

We collected samples from 4 neonates with Bell stage III NEC, 2 neonates with congenital intestinal atresia and 2 convalescent neonates with NEC who served as the controls. Among the NEC cases, infants often developed NEC after 4 weeks of hospitalization, and their birth weight was less than 1500 g (**Table 1**). Serum hypersensitive C-reactive protein (hs-CRP), which is an inflammatory cascade biomarker, was significantly increased in patients with acute NEC. Of note, 1 NEC neonate had intestinal failure, and 3 NEC neonates had neonatal cholestasis (**Table 1**). Next, we investigated whether MD2 plays a role in NEC. Compared with that in the control samples, MD2 expression was significantly increased in clinical samples from NEC neonates according to immunofluorescence analysis (P<0.05) (**Figures 1A, B**).

**Table 1:** Clinical and laboratory data of NEC infants and control volunteers (n=8).

Groups			NEC groups (N1-N4)			Control group (C1-C4)		
Infants	N1	N2	N3	N4	C1	C2	C3	C4
Gestational Age (w+d)	32^+2^	28^+3^	25^+6^	27^+2^	38	39	29^+1^	26^+1^
Birth weight (g)	1380	1260	990	1200	3100	3990	980	740
Age of onset of NEC (d)	29	28	50	37	–	–	10	51
Delivery mode	Caesarean section	Caesarean section	Caesarean section	Vaginal delivery	Vaginal delivery	Vaginal delivery	Caesarean section	Caesarean section
Hs-CRP* in acute phase of NEC (mg/L)	69.48	10.83	67.85	88.56	1.37	1.13	59.88	94.31
Clinical diagnosis	Acute stage of NEC	Acute stage of NEC	Acute stage of NEC	Acute stage of NEC	Congenital anal atresia	Congenital jejunal atresia	Recovery stage of NEC	Recovery stage of NEC
Surgical pathologic staging	NEC III	NEC IIb (multifocal lesions)	NEC IIIa	NEC IIIa	–	–	–	–
Complication	RDS*, PDA*, Neonatal cholestasis, Sepsis, Short bowel syndrome	RDS*, BPD*, PDA*, Sepsis, Neonatal cholestasis, Intraventricular hemorrhage	RDS*, BPD*, PDA*, ROP*, Sepsis, Neonatal cholestasis	RDS*, PDA*, infantile hepatitis syndrome	–	–	RDS*, Sepsis	RDS*, BPD*, PDA*, Sepsis, ROP*, infantile hepatitis syndrome

*hs-CRP: hypersensitive C-reactive protein, RDS: Respiratory distress syndrome, BPD: Bronchopulmonary Dysplasia, ROP: Retinopathy of Prematurity, PDA: Patent ductus arteriosus. The normal range of hs-CRP is less than 8 mg/L. N1-4 stand for patients in NEC group, C1-4 stand for patients in control group.

### Evaluation of the general state, pathological alterations and MD2 protein expression in NEC rats

**Figure 2:**
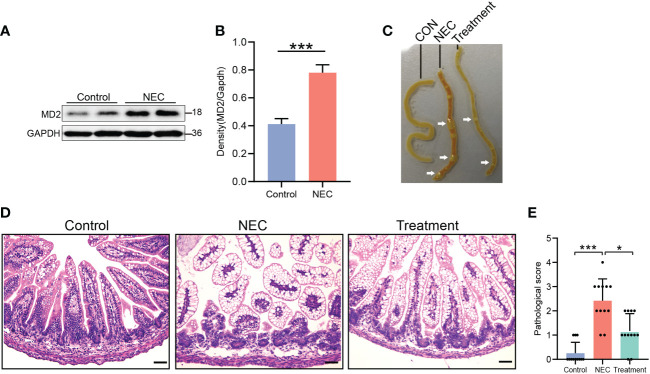
Evaluation of pathological condition, histological score and MD2 protein expression in NEC rats. **(A, B)** The expression of MD2 in the intestinal tissue of NEC neonatal rats was detected by western blot analysis (n=4). The p value for each group comparison using a two-tailed Student’s t test. ***p < 0.001. **(C)** Representative images showing the gross intestinal morphology. **(D, E)** Histological H&E staining of the transverse section of ileal tissue and histological NEC score in each group. Scores of 2 or more were regarded as NEC positive. Scale bar, 50 μm. Data are presented as the mean ± SD (n=12) and analyzed by the Kruskal–Wallis test. *p < 0.05, ***p < 0.001.

During the experiment, NEC neonatal rats gradually developed abdominal distension, diarrhea and yellow mucous stool excretion, and their activity decreased as determined by poor response and drowsiness. In the treatment group, neonatal rats also gradually developed abdominal distension, diarrhea and poor response. In contrast, the treatment group had later occurrence and a lower degree of disease than the NEC group. The general condition of the control and vehicle-treated rats was good, with normal behavior and feces. First, we evaluated the expression of MD2 in the intestinal tissue of NEC rats. As predicted, results from western blot analysis (**Figures 2A, B**) showed that MD2 expression was higher in experimental NEC rats than in control rats. In the NEC group samples, the intestinal tract showed extensive edema, dim coloration with intestinal dilatation, and air within the bowel wall (known as pneumatosis intestinalis), and in severe cases, these effects were accompanied by beaded changes. The intestinal dilatation and pneumatosis of rats treated with MI were mild (**Figure 2C**). Histological examination showed edema in the submucosa and muscular layer, separation of the submucosa and extensive exfoliation of villi in the NEC intestines (**Figure 2D**). Compared with the control rats, the NEC rats presented with a significantly higher grade of histological injury (**Figure 2E**). However, the rats in the treatment group showed a significant decrease in histological injury grade compared to those in the NEC group (**Figure 2E**).

### Blocking MD2 inhibits NF-κB and inflammatory responses in NEC rats

**Figure 3:**
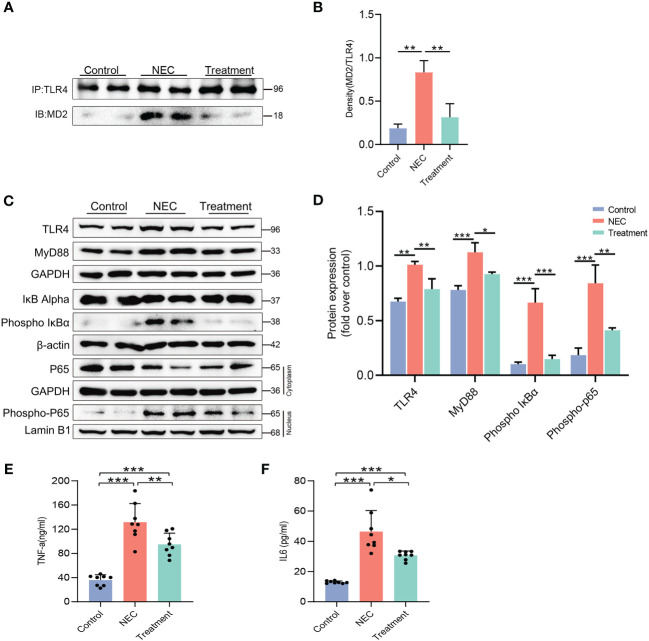
Blocking MD2 inhibits NF-κB and inflammatory responses in NEC rats. **(A, B)** Immunoprecipitation assays showed that MD2 inhibitor pretreatment significantly reduced the formation of the TLR4–MD2 complex in rat intestinal tissue. IB indicates immunoblotting antibody, and IP indicates precipitating antibody (n=3). **(C, D)** Western blot analysis of TLR4 signaling-related proteins, NF-κB activation and nuclear translocation of the p65 subunit of NF-κB (n=3). **(E, F)** TNF-α and IL-6 levels in intestinal tissue lysates were detected by ELISA, and values were normalized to the total protein (n=8). The p value was analyzed by one-way ANOVA with multiple comparisons, followed by Tukey’s *post hoc* test. *p < 0.05, **p < 0.01, ***p < 0.001.

We determined the expression level of the MD2-TLR4 complex by coimmunoprecipitation of rat intestinal tissue. As expected, the level of the MD2-TLR4 complex in the NEC rats was 4 times higher than in the control rats, but the increase was not observed in the treatment group rats (**Figures 3A, B**). The expression of TLR4 and MyD88 in the NEC group was higher than that in the control group, while expression in the treatment group was lower (**Figures 3C, D**). We next evaluated the phosphorylation of IκBα, a step that is necessary for translocation of the NF-κB p65 subunit and activation of NF-κB. The levels of IκBα phosphorylation and NF-κB translocation in the intestinal tissue of the NEC group were significantly increased, while pretreatment with MI significantly inhibited the activation of NF-κB (**Figures 3C, D**). The activation of MD2-TLR4/NF-κB signal transduction prompts the production of proinflammatory cytokines, leading to an inflammatory cascade reaction. ELISA analysis of inflammatory molecules in intestinal tissue showed that the expression of TNF-α (**Figure 3E**) and IL-6 (**Figure 3F**) was upregulated in both the NEC group and the treatment group compared to the control group; however, the levels in the treatment group were lower than those in the NEC group. These results showed that MD2 blockade prevents the activation of MD2-TLR4 signaling and inhibits the production of proinflammatory factors.

### MD2 inhibition attenuates enterocyte apoptosis and intestinal barrier dysfunction in NEC rats

**Figure 4:**
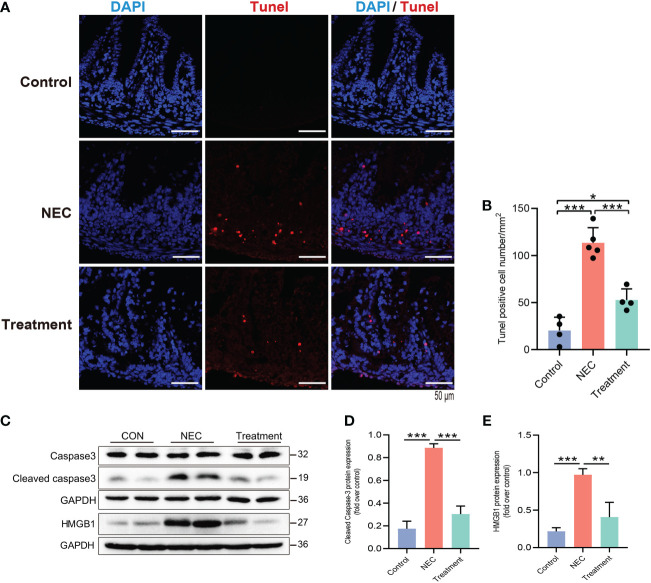
MD2 blockade attenuates apoptosis of intestinal epithelial cells in NEC rats. **(A, B)** Detection of intestinal cell apoptosis in each group of neonatal rats; red represents TUNEL^+^ cells. NEC significantly increased TUNEL^+^ cells, while pretreatment with MD2 inhibitor significantly blocked this effect (n=4 in the control group, n=4 in the treatment group and n=5 in the NEC group). Scale bar, 50 μm. **(C–E)** The protein expression of cleaved caspase-3 and HMGB1 in the intestines of the three groups was analyzed by Western blotting (n=3). The p value was analyzed by one-way ANOVA with multiple comparisons, followed by Tukey’s *post hoc* test. *p < 0.05, **p < 0.01, ***p < 0.001.

Next, we further studied the protective mechanism of MI. Enterocyte apoptosis induced by the TLR4 inflammatory cascade is the central pathway leading to NEC-induced mucosal inflammation. TUNEL staining revealed prominent NEC enterocyte apoptosis, which was significantly attenuated by MI (**Figures 4A, B**). Caspase-3 is a marker for apoptosis, and its degree of cleavage can reflect the level of apoptosis. The increased expression of the protein HMGB1 can reflect an increase in tissue or cell injury. As shown in **Figures 4C–E**, the expression levels of cleaved caspase-3 and HMGB1 were highly increased in NEC, while the expression levels of cleaved caspase-3 and HMGB1 were markedly suppressed by MI.

**Figure 5:**
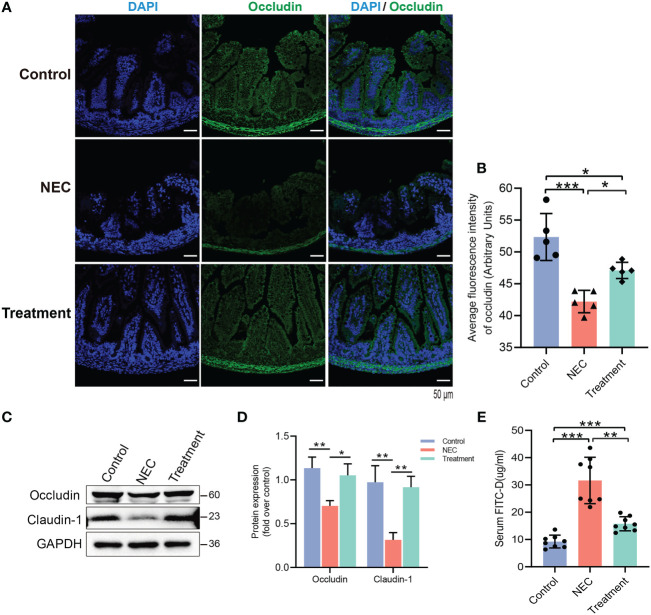
Evaluation of the effect of MD2 on intestinal mucosal barrier function in NEC rats. **(A, B)** Occludin expression in the intestinal tissue of rats. Occludin (green) and DAPI staining of nuclei (blue). The scatter plots represent the mean value of the average optical density in 3 random fields in each section of the quantification diagram. Scale bar, 50 μm, n=5. **(C, D)** The protein expression of occludin and claudin-1 in the intestines of the three groups was analyzed by Western blotting (n=3). **(E)** The intestinal permeability was detected by FITC-D (n=8). The p value was analyzed by one-way ANOVA with multiple comparisons, followed by Tukey’s *post hoc* test. *p < 0.05, **p < 0.01, ***p < 0.001.

We further evaluated the intestinal barrier function of neonatal rats. The expression of occludin protein in the NEC group was decreased, while the expression of occludin protein in the control and treatment groups was relatively high and evenly distributed (**Figures 5A, B**). In the NEC group, the levels of tight junction (TJ) proteins, including claudin-1 and occludin, were decreased (**Figures 5C, D**), thereby leading to impairment of the intestinal barrier function. MD2 blockade protected against this loss of function caused by NEC (**Figures 5A–D**). The permeability of the intestinal mucosa was detected by FITC-D. The intestinal permeability of FITC-D was significantly enhanced in NEC rats compared to control rats, while the leakage of FITC-D into circulation was reduced in rats pretreated with MI (**Figure 5E**).

### Efficiency of MD2 overexpression or knockdown and effects of LPS on NF-κB and the inflammatory response in IEC-6 enterocytes

**Figure 6:**
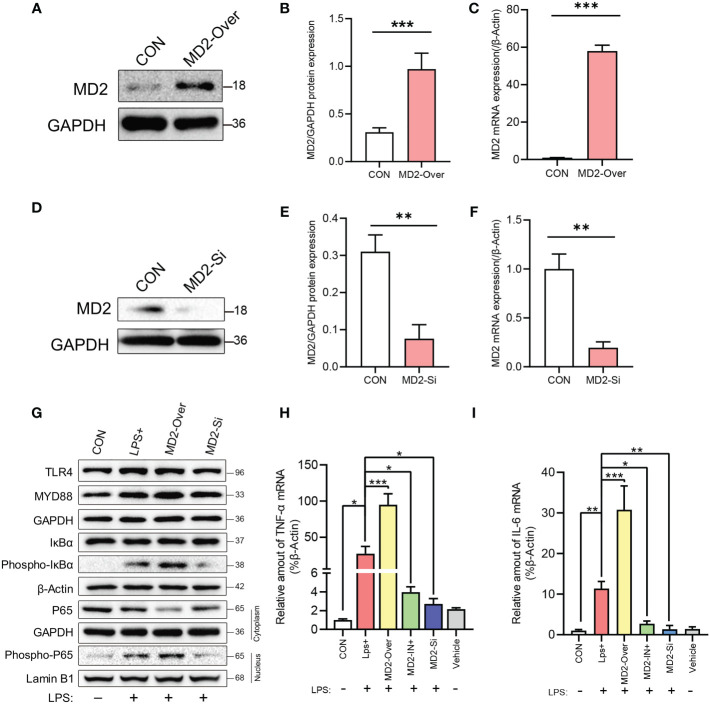
Efficiency of MD2 overexpression or knockdown and the effects of LPS on NF-κB and the inflammatory response in IEC-6 enterocytes. **(A–C)** Evaluation of MD2 overexpression efficiency by western blot analysis (n=4) and RT–qPCR analysis (n=3). **(D–F)** Evaluation of MD2 silencing efficiency (n=3). **(G)** Effects of LPS on TLR4 signaling-related proteins, NF-κB activation and nuclear translocation of the p65 subunit of NF-κB in IEC-6 enterocytes (n=3). **(H, I)** The effect of LPS on proinflammatory cytokines in IEC-6 enterocytes was analyzed by RT–qPCR (n=3). LPS+, IEC-6 + LPS; MD2-Over, MD2-overexpressing IEC-6 + LPS; MD2-IN-1+, IEC-6 + MD2 inhibitor + LPS; MD2-Si, MD2-deficient IEC-6 + LPS; Vehicle, IEC-6 treated with DMSO at a 1:1000 dilution. **(B–F)** p value for each group comparison using a two-tailed Student’s t test. **p < 0.01, ***p < 0.001. **(H, I)** The p value was analyzed by one-way ANOVA with multiple comparisons, followed by Tukey’s *post hoc* test. *p < 0.05, **p < 0.01, ***p < 0.001.

We next used a lentivirus-mediated stable MD2 overexpression cell line (IEC-6), with the MD2 gene silenced by siRNA, to further explore the effect of MD2 on IEC-6 enterocytes stimulated by LPS. Transfection of the lentiviral plasmid expressing MD2 shRNA resulted in significantly higher MD2 protein and mRNA levels in IEC-6^MD2-Over^ cells than in IEC-6-CON cells (**Figures 6A–C**). After transfection with siRNA, the mRNA and protein levels of MD2 in IEC-6^MD2-Si^ cells were significantly downregulated compared to those in nontarget-treated cells, and the effective silencing of MD2 expression was 80.5% (**Figures 6D–F**).

After LPS induction, the levels of IκBα phosphorylation and NF-κB P65 nuclear translocation in IEC-6^MD2-Over^ cells were upregulated compared to those in control cells, while the activation of NF-κB was decreased in IEC-6^MD2-Si^ cells (**Figure 6G**). RT–qPCR analysis indicated that LPS significantly increased the expression of TNF-α and IL-6 mRNA in IEC-6^MD2-Over^ cells compared to IEC-6-CON cells, and this increase in TNF-α and IL-6 mRNA expression was prevented by MD2 gene silencing and MI pretreatment (**Figures 6H, I**).

### Effects of MD2 on inflammatory injury and barrier function in IEC-6 enterocytes

**Figure 7:**
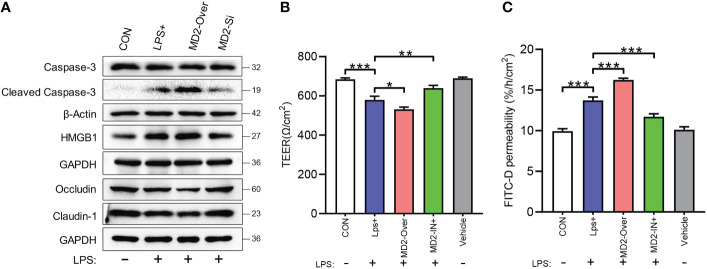
Effects of MD2 on inflammatory injury and barrier function in IEC-6 enterocytes. **(A)** The expression of HMGB1, caspase-3, cleaved caspase-3, occludin and claudin-1 was measured by western blot analysis (n=3). **(B)** Cell monolayer permeability was detected using TEER. **(C)** Determination of FITC-D permeability (n=3). LPS+, IEC-6 + LPS; MD2-Over, MD2-overexpressing IEC-6 + LPS; MD2-IN-1+, IEC-6 + MD2 inhibitor + LPS; Vehicle, IEC-6 treated with DMSO at a 1:1000 dilution. p value was analyzed by one-way ANOVA with multiple comparisons, followed by Tukey’s *post hoc* test. *p < 0.05, **p < 0.01, ***p < 0.001.

As shown in **Figure 7A**, LPS stimulation highly activated the inflammatory injury-related protein HMGB1 and the apoptosis-related protein caspase-3 in IEC-6 enterocytes. Moreover, MD2 overexpression further increased this activation, whereas MD2 silencing reduced this activation. After LPS-induced injury, the expression of claudin-1 and occludin in IEC-6^MD2-Over^ cells was significantly decreased compared to IEC-6-CON cells and IEC-6^MD2-Si^ cells (**Figure 7A**). TEER is a common method to detect monolayer cell barriers and evaluate the integrity of cell−cell tight junctions. The TEER assay showed that the TEER value of monolayer cells decreased after IEC-6 cells were injured by LPS. The most severe decrease in TEER value was seen in IEC-6^MD2-Over^ cells, while pretreatment with MI suppressed the decrease in TEER value (**Figure 7B**). Notably, the decrease in TEER value was accompanied by an increase in FITC-D fluorescence intensity. FITC-D detection showed that the cell permeability of IEC-6^MD2-Over^ cells was higher than that of IEC-6 cells with or without LPS intervention, while the cell permeability of IEC-6 cells pretreated with MI was diminished (**Figure 7C**).

## Discussion

NEC is a destructive and inflammatory intestinal disease that affects the life of premature infants and is characterized by multiple intestinal injuries ranging from epithelial injury to transmural involvement and perforation (2, 37). The role of TLR4 in the pathogenesis of neonatal NEC has become increasingly important. Excessive stimulation of TLR4 in the intestinal epithelium leads to NEC by initiating the signal cascade reaction and further leading to breakdown of the intestinal barrier (15, 37–39). Activated TLR4 triggers the activation of downstream signaling pathways and leads to the translocation of NF-κB, leading to the overexpression of proinflammatory molecules (40). Excessive inflammatory mediator damage and necrotic intestinal tissue destroy the mucosal barrier, resulting in the development of NEC (41). Of note, apoptosis of enterocytes is the precursor of intestinal mucosal injury in NEC (42).

MD2 can directly recognize and bind to the LPS lipid A domain with the activation of TLR4, which is an indispensable auxiliary protein (43). MyD88 is the main downstream effector of the TLR4-MD2-LPS complex following binding to LPS (44). The present study found that MD2 is highly correlated with NEC in preterm infants. Previous studies have shown that the transcription level of the MD2 gene in acute-phase intestinal tissues of NEC patients was increased (21). Similarly, we confirmed that the MD2 protein was upregulated in the acute phase of neonatal NEC. Formula feeding, hypothermia, and asphyxia have been identified as risk factors for the development of NEC (45–47). In this work, the neonatal rat NEC model was constructed by targeting multiple factors in the pathogenesis of NEC using formula feeding and hypoxia-cold stimulation with reference to previous studies (48–51). This model has been proven to be an ideal NEC modeling method that reproduces the typical pathophysiological changes in NEC by combining a variety of pathogenic factors. In this experiment, NEC rats had NEC-like lesions similar to those observed in clinical NEC patients, including abdominal distension, gastric retention, diarrhea, intestinal dilatation, inflammatory leukocyte infiltration, necrosis and perforation. Pretreatment with MI reduced the incidence and severity of NEC and prevented intestinal inflammatory injury caused by hypoxia/cold stress, determined by the decrease in NEC scores and the levels of TNF-α and IL-6 in the ileum. A large amount of evidence shows that there are high concentrations of LPS and TLR/MyD88-associated proinflammatory cytokines in the serum of NEC patients, including IL-1β, IL-6 and TNF-α (52, 53). MI administration in experimental NEC rats preserved the intestinal villous architecture, maintained barrier function and significantly decreased apoptosis levels. During initiation of the inflammatory cascade reaction, rapid degradation of IκB-α in the cytoplasm and downstream translocation of phosphorylated NF-κB p65 are necessary to activate NF-κB. In this study, both of these processes were attenuated by pretreatment with MI. Thus, the findings presented here showed that MI prevented NEC-related intestinal injury in neonatal rats by targeting the MD2-TLR4/NF-κB inflammatory pathway.

Barrier dysfunction is another important pathophysiological basis of NEC. During the early stages of NEC development, abnormal cell death and epithelial barrier disruption are necessary conditions for further development (11, 15, 16). Numerous studies have demonstrated that the intestinal barrier dysfunction observed in human NEC is characterized by the downregulation of mucins and TJ proteins, such as zonula occludens-1, occludin and claudin-4 (54–56). In this study, we found that the leakage of FITC-D into the circulation increased and the expression of claudin-1 and occludin decreased in NEC samples, suggesting severe barrier dysfunction. The intestinal epithelial (enterocyte) cell line IEC-6 is often used as an NEC model system *in vitro* (17, 57–59). IEC-6 cells are nontransformed intestinal cells that have abundant expression of TLR4 and respond to LPS. These cells are derived from rat small intestine with epithelioid morphological characteristics and an immature crypt-like phenotype (60). The IEC-6 model induced by LPS *in vitro* showed that overexpression of MD2 stimulated damage to the single-layer cell barrier, while both MD2 silencing and MI pretreatment were protective. Previous studies have shown that suppression of the TLR4 signaling pathway protects the intestinal barrier function of NEC (61–63). The barrier-protective effect of MD2 has been previously reported in mice with chronic colitis (29). Barrier dysfunction caused by severe intestinal epithelial cell death is the basis of intestinal mucosal injury in NEC, in which TLR4 plays a key role (17, 64). In the present study, we found that enterocyte apoptosis increased and that caspase-3 and HMGB1 were highly activated in NEC rats, and these effects were reversed by pretreatment with MI. HMGB1 is an important proinflammatory cytokine and inflammatory transmitter (65–67). The increased expression of HMGB1 leads to enhancement of the inflammatory response in the tissue and promotes the development of NEC (68, 69).

The present study showed that targeting MD2 to block the activation of TLR4 reduces excessive inflammatory storms, intestinal barrier function injury and intestinal cell apoptosis in NEC patients. The present study had several limitations. The number of human samples was small, and the direct effect of MD2 on TJs was not explored in depth. In addition, genetically engineered rats and human intestinal epithelial cell lines were not used to further explore the mechanism. The specificity of the treatment window that was explored is another limitation of this study. In future work, we will further explore the appropriate time course of treatment.

## Conclusion

In summary, we found that the MD2 protein is upregulated in NEC infants. MD2 leads to overactive inflammation through the MD2-TLR4/NF-κB signaling axis and promotes enterocyte apoptosis and barrier dysfunction, thereby exacerbating NEC. Because TLR4 activation is a common pathogenic alteration of NEC, inhibiting MD2 is a potential therapeutic target for the treatment of NEC.

## Data availability statement

The original contributions presented in the study are included in the article/**Supplementary Materials**. Further inquiries can be directed to the corresponding author.

## Ethics statement

All the studies followed the guidelines for the ethical treatment of human specimens and were approved by the Ethics Committee of Guangzhou Women and Children's Medical Center (Ethical approval number: [2022] 059A01). According to the Declaration of Helsinki, written informed consent was obtained from the parents of all the neonates. All animal experiments were approved by the Institutional Animal Care and Use Committee of Guangzhou Medical University and performed according to the institutional guidelines.

## Author contributions

DH, WZ, YT and MZ contributed to the study design. DH, YL and JC performed experiments. DH and PW analyzed data. WZ performed and/or contributed critically to all experiments. DH and PW drafted manuscript. PW and WZ edited and revised the manuscript. All the authors have read and approved the final manuscript.

## Funding

Guangzhou Science and Technology Plan Project (No. 201904010484 to WZ, No. 202102080247 to PW). Basic and Applied Basic Research Foundation of Guangdong Province (No. 2022A1515012354 to PW).

## Acknowledgments

We thank Professors WZ and YT for providing reagents and help. The authors also thank all the staff of the Yaping Tang Experimental Research Group Unit of the Institute of Pediatrics, Guangzhou Women and Children’s Medical Center.

## Conflict of interest

The authors declare that the research was conducted in the absence of any commercial or financial relationships that could be construed as a potential conflict of interest.

## Publisher’s note

All claims expressed in this article are solely those of the authors and do not necessarily represent those of their affiliated organizations, or those of the publisher, the editors and the reviewers. Any product that may be evaluated in this article, or claim that may be made by its manufacturer, is not guaranteed or endorsed by the publisher.
