# Supplementary figures and images for: Selective targeting of MD2 attenuates intestinal inflammation and prevents neonatal necrotizing enterocolitis by suppressing TLR4 signaling
Source: Front Immunol. 2022 Nov 1;13:995791. doi: 10.3389/fimmu.2022.995791 (PMC9663461; doi:10.3389/fimmu.2022.995791)

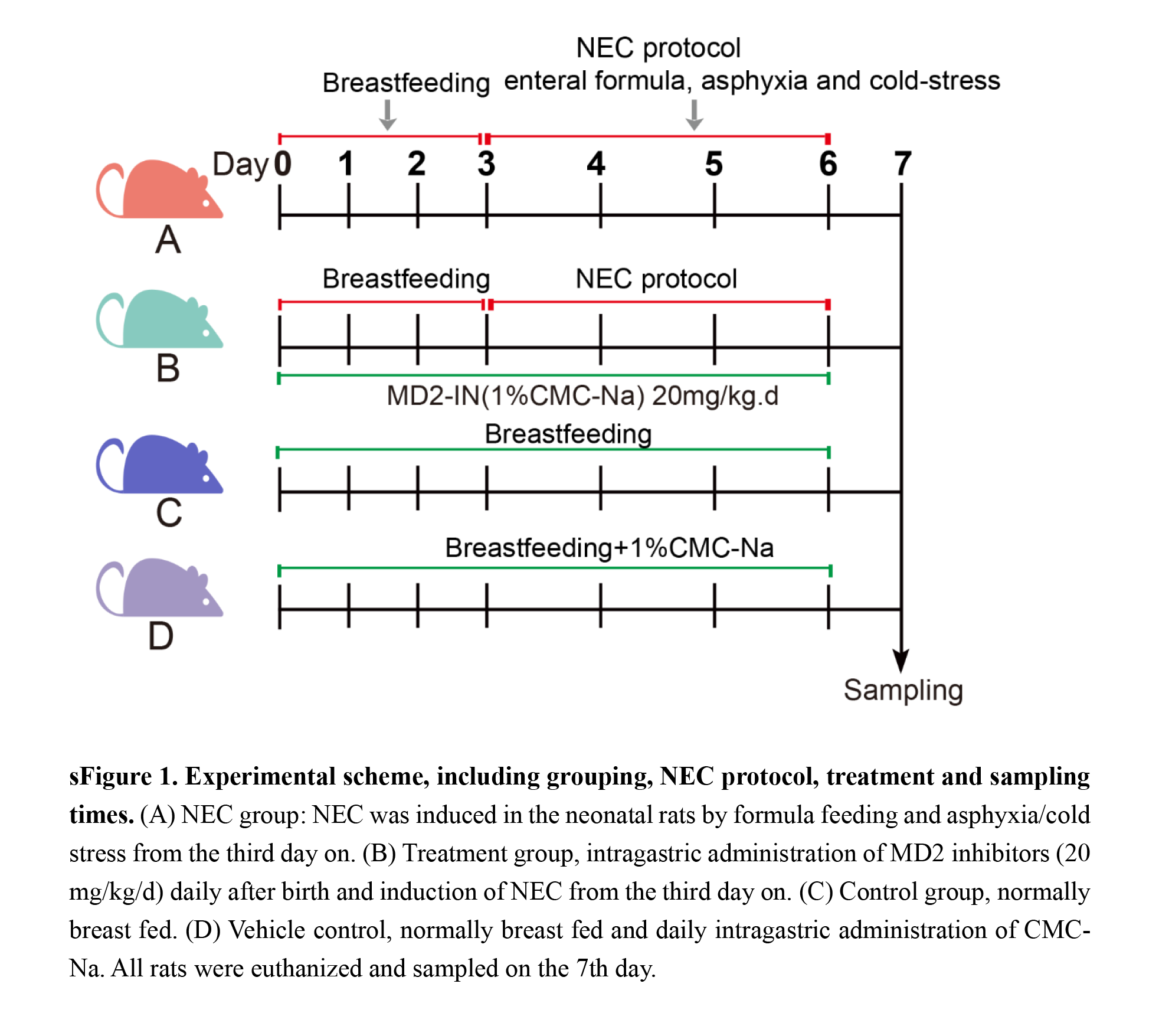

Supplement: Supplementary file 1 [file Image_1.tif]
